# Support Needs of Patients with Cushing's Disease and Cushing's Syndrome: Results of a Survey Conducted in Germany and the USA

**DOI:** 10.1155/2018/9014768

**Published:** 2018-10-09

**Authors:** Ilonka Kreitschmann-Andermahr, Sonja Siegel, Christa Gammel, Karen Campbell, Leslie Edwin, Agnieszka Grzywotz, Victoria Kuhna, Maria Koltowska-Häggström, Oliver Müller, Michael Buchfelder, Bernadette Kleist

**Affiliations:** ^1^Department of Neurosurgery, University of Duisburg-Essen, 45147 Essen, Germany; ^2^Department of Neurosurgery, University Hospital Erlangen, 91054 Erlangen, Germany; ^3^Cushing's Support and Research Foundation, Plymouth, MA 02360, USA; ^4^Department of Neurosurgery, Ev. Hospital Oldenburg, 26121 Oldenburg, Germany; ^5^Department of Women's and Children's Health, Uppsala University, Akademiska Sjukhuset, SE-751 85 Uppsala, Sweden

## Abstract

**Background:**

Cushing's disease (CD) and Cushing's syndrome (CS) are chronic illnesses, characterized by symptoms of prolonged hypercortisolism, which often changes to hypocortisolism after successful treatment. In view of the high disease burden of CD/CS patients and long-term impaired quality of life, the present survey was conducted to gain information about subjective illness distress and patients' specific needs in terms of supportive measures beyond medical interventions.

**Patients and Methods:**

Cross-sectional questionnaire study including patients with CD treated in 2 German neurosurgical tertiary referral centers and CD/CS patient members of a US-based patient support group completed a survey inquiring about disease burden, coping strategies, and support needs. Additionally, the degree of interest in different offers, e.g., internet-based programs and seminars, was assessed.

**Results:**

84 US and 71 German patients answered the questionnaire. Patients in both countries indicated to suffer from Cushing-related symptoms, reduced performance, and psychological problems. 48.8% US patients and 44.4% German patients stated that good medical care and competent doctors helped them the most in coping with the illness. US patients were more interested in support groups (*p* = 0.035) and in courses on illness coping (*p* = 0.008) than the German patients, who stated to prefer brochures (*p* = 0.001). 89.3% of US patients would attend internet-based programs compared to 75.4% of German patients (*p* = 0.040). There were no differences between groups for the preferred duration of and the willingness to pay for such a program, but US patients would travel longer distances to attend a support meeting (*p* = 0.027).

**Conclusion:**

Patients in both countries need skilled physicians and long-term medical care in dealing with the effects of CD/CS, whereas other support needs differ between patients of both countries. The latter implies that not only disease-specific but also culture-specific training programs would need to be considered to satisfy the needs of patients in different countries.

## 1. Introduction

Cushing's disease (CD) and Cushing's syndrome (CS) are illnesses characterized by symptoms due to prolonged exposure to elevated cortisol levels, which are associated with increased morbidity and mortality from metabolic, musculoskeletal, infectious, thrombotic, cardiovascular, and neuropsychiatric complications [1, 2]. In CD, the cause of the hormone excess is an adrenocorticotrophic hormone- (ACTH-) secreting pituitary adenoma, which stimulates adrenal cortisol secretion [1]. In CS, the reason for hypercortisolism may be exogenous (i.e., prolonged glucocorticoid treatment) or endogenous, such as a benign or malignant cortisol-secreting tumor of the adrenal gland or paraneoplastic ACTH secretion [3, 4]. With an incidence of 2–3/million, CS and CD are rare diseases [5–7].

Clinical signs and symptoms of Cushing's include rapid weight gain, plethora, easy bruising, edema, proximal limb muscle fatigue, impaired glucose tolerance, mood disorders, such as depression and anxiety, cognitive difficulties, osteoporosis, and cardiovascular problems [1, 4, 8–10]. First-line therapy is the surgical removal of the hormone-secreting tumor and, in cases of iatrogenic CS, lowering the dose of or discontinuing glucocorticoids if possible. If hypercortisolism cannot be normalized by these measures, radiotherapy, steroid-lowering medications, or bilateral adrenalectomy may also be employed in patients with endogenous CS and CD [11, 12]. However, next to other therapeutic side effects, treatment of CS and CD often leads to hypocortisolism necessitating hydrocortisone replacement therapy and bearing the potential complication of life-threatening Addisonian crisis [13].

On the basis of the above, it becomes immediately clear that CS and CD are chronic diseases that may not be easily cured and have long-term effects on patients' health, appearance, well-being, and quality of life (QoL). Studies on patients with Cushing's confirm that patients' QoL can be impaired years after successful treatment even though the disease itself may be well-controlled or in long-term remission [9, 14–16].

In recent years, restoration of QoL in patients with chronic diseases has become an increasingly important treatment goal in clinical practice, resulting in the implementation of special support programs for many chronic diseases ranging from cancer and multiple sclerosis to diabetes mellitus [17–19]. It was, therefore, the aim of the present study to explore the disease burden and unmet support needs of patients with Cushing's against the background of developing strategies for targeted patient support beyond medical interventions.

## 2. Patients and Methods

### 2.1. Patients

This cross-sectional patient-reported survey was conducted among adult (age ≥ 18 years) patients with CD who had undergone pituitary surgery for biochemically proven CD in two German neurosurgical university centers (Erlangen-Nuremberg and Duisburg-Essen) and patients with CD or CS who are members of the US-based Cushing's Support and Research Foundation (CSRF) and who also had already received diagnosis and treatment of CD/CS. Inability to fill in the survey was the only exclusion criterion.

### 2.2. Survey/Questionnaire Description

A patient-reported outcome survey (PRO survey) was developed to assess information about (1) the current burden of Cushing-related symptoms, (2) time points when support was needed the most, (3) factors that have helped the patients the most in coping with the disease, and (4) disease-specific support needs, interest in a support program, and topics of interest. These questions were compiled based on the results of former research by our group [10] and the current state of research on QoL and coping in patients with CD and CS [9, 14–16]. A neurologist (IKA), a neurosurgeon (MB), a psychologist (SoS), and, for the US version of the questionnaire, one of the former directors (KC) of the CSRF developed the survey.

The survey comprised 14 questions to be answered based on given response options or as a free-text. The interest of the participants in a support program was assessed using a 5-point Likert scale, using the statements: not at all, a little, moderate, a lot, and absolutely. The statements were coded from 1 (not at all) to 5 (absolutely) for statistical analyses. The questionnaire was developed in German, and for the US participants was translated into English by a state-certified translator (IKA) and by a native English speaking neurosurgeon in training (VK) and checked by KC to ensure comprehension of the translated questions.

For easier handling, the questionnaire was then programmed as an online-based survey that could be filled in by the US patients via an activation link for a homepage sent by e-mail via the CSRF. The homepage was operated by the University Hospital Essen (Germany) and was hosted on a secure server of the hospital. The German patients received the paper-based version by mail.

The study was conducted in accordance with the Guideline for Good Clinical Practice and the Declaration of Helsinki [20, 21]. All patients gave informed consent, and the study was approved by the Ethics Committee of the University of Duisburg-Essen.

### 2.3. Statistical Analysis

Statistical analysis was conducted using SPSS Statistics 23 (Statistical Package for the Social Sciences, IBM, Armonk, USA). Descriptive data of the German and the US group are displayed as frequency and valid percent or as mean ± standard deviation. For group comparison, metric variables were tested for normality using the Shapiro-Wilk test in addition to Q-Q-plots. In cases of nonnormality of data, the Mann–Whitney *U* test instead of Student's *t*-test was used. Nominal data were compared by chi-square test or, if expected frequencies were below five, Fisher's exact test. Answers provided in free-text fields were clustered and counted (thematic qualitative analyses). A *p* value of <0.05 was considered as statistically significant.

## 3. Results

84 US and 71 German patients answered the questionnaire. There was no difference between groups with regard to sex and age (*p* > 0.05, Table 1).

### 3.1. Disease Burden

In response to the question “Which aspects of your Cushing's condition bother/bothered you the most?” which assessed current or past disease burden, patients reported a variety of aspects which could be clustered into four major symptom groups that are displayed in Table 2 (answers provided in a free-text field), together with a selection of the participants' answers in their own words. Comments about the length of the diagnostic process were only provided by US patients. The US patients were bothered more by common symptoms of Cushing's and reduced performance, while the German patients were bothered to a greater extent by psychological impairment (descriptive representation).

### 3.2. Coping Strategies

**Figure 1:**
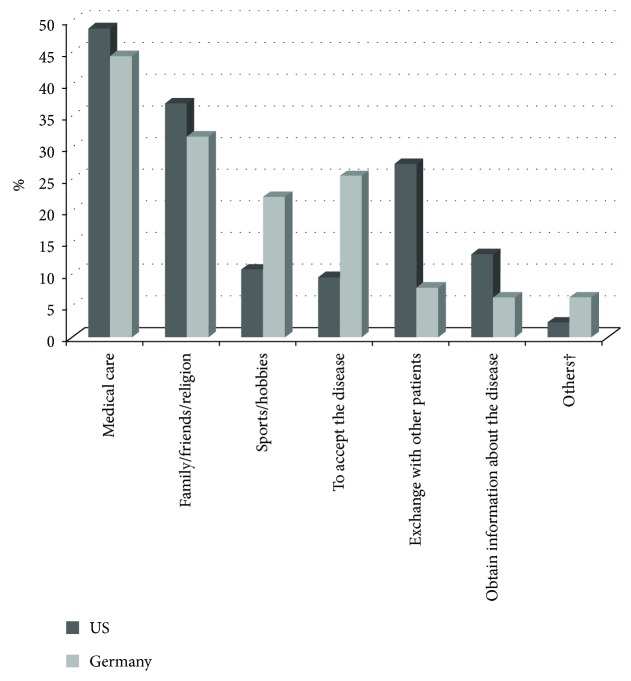
Factors that helped the patients the most in coping with the illness. Answers were provided in a free-text field and were clustered and counted. †Others are nothing helped and time off work.

48.8% of patients from the US and 44.4% of the German patients stated that good medical care and competent, skilled doctors were of major help in coping with Cushing's, followed by the support of family/friends and religion (US: 36.9% vs. Germany: 31.7%). Sports and hobbies (US: 10.7% vs. Germany: 22.2%) as well as accepting the disease (US: 9.5% vs. Germany: 25.4%) were used more often by German patients as coping strategies to deal with the disease, whereas the exchange with other patients (US: 27.4% vs. Germany: 7.9%) and to obtain information about the disease (US:13.1% vs. Germany: 6.3%) were more important to the US patients (Figure 1).

### 3.3. Support Needs

Support needs of the patients are displayed in Table 3. Support was needed to a greater extent before therapy by the U.S patients (*p* = 0.035). 12.7% of the German patients stated not to have wanted further support at any time of their illness, compared to only 2.4% of the US patients (*p* = 0.024). On the other hand, US patients were more interested in support groups, in courses on illness coping, in seminars and workshops, and educational offers for the family than the German patients (all *p* < 0.05), who stated to prefer brochures (*p* = 0.001).

The general interest (assessed by Likert scale) in a specific support program for patients with Cushing's was higher in US patients (3.8 ± 1.10) than in German patients (3.1 ± 1.26) with *p* = 0.001. 28 (33.3%) of the US patients but only 11 (15.9%) of the German patients stated a very strong interest, and 21 (25.0%) of the US patients and 15 (21.7%) of the German patients stated a considerable interest in such a program. Nine (13.0%) German and 2 (2.4%) US patients expressed no interest in a support program.

In regard to specific topics that should be covered by a support program, more US patients than German patients wished to communicate with other patients (*p* = 0.006) and to learn more about stress management (*p* = 0.001; Table 4). 89.3% of US patients would attend internet-based programs compared to 75.4% of German patients (*p* = 0.040). There were no differences between groups for the preferred duration of and the willingness to pay for such a program, but US patients would be willing to travel longer distances to attend a support meeting (*p* = 0.027; Table 4).

## 4. Discussion

To our knowledge, this is the first patient-reported survey which demonstrates the need for additional support apart from medical interventions in patients with CD or CS, although the negative long-term impact of these illnesses on health-related QoL has been confirmed in many studies [22–27]. Patients in both Germany and the USA who completed our survey reported a wide spectrum of past or present Cushing-related symptoms, psychological impairment, and reduced physical and mental performance. In line with the high subjective illness distress, the vast majority of the participants (97% in the USA, 87% in Germany) stated that they wanted additional support at any time during the disease, most often after diagnosis and during the first year of treatment. Many of them were willing to pay for such additional support.

This snapshot is in accordance with themes discussed in focus groups of pituitary adenoma patients and conducted by a Dutch research group, where mood problems, negative illness perceptions, and issues of physical, cognitive, and sexual functioning were among the most prominent complaints of patients with CD [28]. Based on the feedback provided by their focus group participants, the research group developed and validated a questionnaire for pituitary patients, which aims to assess to what extent patients are bothered by consequences of the disease as well as their needs for support [29]. However, results on the practicability and usefulness of this questionnaire and the consequences drawn thereof in clinical practice are yet to be awaited.

The Dutch researchers' focus group results and the feedback given by our surveyed patients reflect unmet support needs despite receiving medical care in modern western healthcare environments. Patients with CD and CS carry the burden of their illness that often develops insidiously and may remain undiagnosed for a long time and causes physical disfigurement and severe comorbidities. The illness may not necessarily become controlled after surgical and/or medical intervention. Moreover, by the nature of hypercortisolism in active and hypocortisolism in treated disease, CD and CS are prone to be accompanied by mental symptoms such as depression and anxiety [30, 31]. Such a course of illness requires constant adaptation and possibly changes of patients' healthcare management to control symptoms, which can cause patients to experience stress and uncertainty [32].

In some respects, modern health care systems have already acknowledged the additional support needs of chronically ill patients. The insight, indicating that a model of care, where the patient is seen as the recipient and the physician as the giver of medical care, does not suit the needs and reality for most patients with chronic illnesses. This has led to the development of new models of care in which patients move from a passive role as healthcare recipients towards an active role as equally important partners in the management of their illness. The Chronic Care Model (CCM) developed by Wagner et al. in 2001 [33] is such a model, according to which high-quality chronic illness care involves collaborative, productive interactions between active and well-informed patients and multidisciplinary teams of healthcare providers on the topics of illness assessment, optimization of therapy, and follow-up as well as self-management support [34]. It has been estimated that 70–80% of people living with chronic illness could reduce their illness burden and costs by appropriate self-management [35]. For such reasons, the improvement of patients' abilities in the self-management of their illness is a major component of patient support programs. Such programs have already been implemented for patients with cancer, multiple sclerosis, diabetes mellitus, chronic back pain, and other diseases [18, 19, 36–42]. Many of these programs are well received by the respective patient populations and have demonstrated a high degree of effectiveness in terms of better health outcomes, QoL, and functional status [43–45]. A recent definition of health has even acknowledged the importance of self-management in chronic diseases by defining health as “the ability to adapt and self-manage in the face of social, physical, and emotional challenges” [46].

The multitude of reported positive effects, ranging from improved clinical and psychosocial outcome over better adherence and self-management to decreased health care costs, encourages the development of specific support programs for CD/CS [17, 47, 48]. However, in the case of CD and CS, the development of special programs devoted solely to this patient group is likely to be cost-intensive and, due to the rareness of the disease, beneficial to only very few patients. Yet, despite the unique features of their illness, patients with Cushing's do not differ in all respects from other patients with chronic conditions such as heart disease, multiple sclerosis, or diabetes. Common challenges associated with the management of such conditions include dealing with symptoms and disability, managing complex medication regimes, having to make lifestyle changes, maintaining a proper diet and exercise, adjusting to psychological and social demands, and engaging in effective interaction with their health care professionals. A study by our group has shown that negative coping strategies are a major determinant of poor QoL, depression, and embitterment in patients with CD [10]. Since many techniques like learning adaptive coping strategies, rules for healthy nutrition, or basic exercises are universally useful, the adaption of already existing programs from other diseases might be a sensible first step for the establishment of self-management programs for patients with Cushing's. Such an effort has already been made for patients with pituitary disease in general by a Dutch research group, which implemented and evaluated a “Patient and Partner Education Programme for Pituitary Disease” (PPEP-Pituitary) [33]. They found positive effects of this program in patients and partners and concluded that future research should focus on the refinement and implementation of such a self-management program into clinical practice.

Our results suggest that such a program should focus on the time before and the first year after treatment, which due to the sudden cortisol deprivation is severely stressful for the patient. Topics worth covering might be communication, nutritional advice, and exercise, since these are the topics most frequently requested in both countries. Stress management seems to be of interest especially to US patients. Also, the setting should be adapted to cultural preferences. In the USA, in-person support groups are highly desired with patients willing to travel considerable distances and pay for such programs, while German patients seem to prefer written information in leaflets or brochures. Patients in both countries express interest in web-based forms of support.

Last but not the least, our results underline the importance of patient support groups that are already in place. Already a quarter of the US patients report that the exchange with other patients was most helpful to them. Nevertheless, 50% express a wish for more patient support groups. It can be speculated that some patients might not know about already existing groups. Oftentimes, it might already improve a patient's well-being to ensure that he has access to all the existing support like support groups or information material.

One limitation of the present study is that the use of patient response tools such as a PRO survey may have introduced a bias as it can be speculated that patients with a higher disease burden are more likely to participate in a survey querying disease symptoms and need for support. Nevertheless, the results must be understood as a call to identify, implement, and evaluate valid support programs with an emphasis of self-management for patients with Cushing's and other endocrine diseases, preferably in a multicenter setting. Culture-specific support needs should also be taken into account.

## 5. Conclusion

Patients with Cushing's in the USA and Germany not only need competent physicians and long-term medical care in dealing with the effects of CD/CS but also request additional support besides medical interventions, while the interest in specific topics addressed in support programs differs somewhat between patients of both countries. The latter implies that not only disease-specific but also culture-specific training programs would need to be considered to satisfy the needs of patients in different countries.

## Figures and Tables

**Table 1:** Characteristics of the study population for both groups. Data are presented as frequency (*n*) and valid percent (%) or mean ± standard deviation.

Variable	US (*n* = 84)	Germany (*n* = 71)	*p* value
*Sex*			
Female	77 (91.7)	59 (83.1)	0.141
Male	7 (8.3)	12 (16.9)	
*Age (years)*	50.1 ± 13.73	48.8 ± 13.64	0.555
*Reason for hypercortisolism*			
Pituitary adenoma	56 (66.7)	71 (100.0)	
Adrenal tumor	21 (25.0)	—	
Others^†^	7 (8.3)	—	

^a^Fisher's exact test. ^†^Other reasons are pursuing ectopic or pituitary (*n* = 1), bilateral macronodular adrenal hyperplasia (*n* = 1), ectopic ACTH syndrome (*n* = 2), and steroid treatment (*n* = 3).

**Table 2:** Answers of the patients in regard to the question “Which aspects of your Cushing condition bother/bothered you the most?” Answers were clustered and counted. Data are presented as frequency (*n*) and valid percent (%).

Variable	US (*n* = 84)	Germany (*n* = 65)
*Common symptoms related to cortisol overproduction*	71 (84.5)	34 (52.3)
Moon face, buffalo hump, red cheeks, bruising transparent skin, inability to lose weight, acne, high blood pressure, heart racing, cardiopulmonary effects, hair growth, dizziness, body pain, swelling of body/limbs, sweating		
*Reduced performance*	39 (46.4)	24 (36.9)
Muscle weakness, muscle loss, morning insomnia, fatigue, low energy		
*Psychological impairment*	51 (60.8)	43 (66.2)
Being depressed, depression, anxiety, panic attacks, nervous, scared, tantrums, sexual dysfunction, feeling crummy, cognitive changes, neg. effects on self-confidence, confused in large crowds/noisy places, felt like I was on psychotropic drugs and slipping between dimensions, being cyclical, worry about residual effects on my brain, foggy brain, memory and attention issues, brain always racing		
*Diagnostic process*	8 (9.5)	—
Fighting for diagnosis; the doctors' inability to link all of these symptoms; was asked if I needed counseling for my obsession with my health; how long it took for a diagnosis; I suffered for years at the hand of doctors; MDs not listening and saying stupid things like I was stressed out		

**Table 3:** Support needs of the patients. Data are presented as frequency and valid percent (%).

Variable	US (*n* = 84)	Germany (*n* = 71)	*p* value
*Stages at the course of the disease patients wished for more support (multiple answers possible)*			
Before therapy	53 (63.1)	32 (45.1)	**0.035**
In weeks directly after therapy	36 (42.9)	26 (36.6)	0.511
Within the first year after the start of therapy	38 (45.2)	30 (42.3)	0.747
Never	2 (2.4)	9 (12.7)	**0.024**
			—
*Current interest in different kinds of supportive offers (multiple answers possible)*			
In-person support group	43 (51.2)	24 (33.8)	**0.035**
Webinar/online forum	45 (53.6)	28 (39.4)	0.106
Lectures	29 (34.5)	31 (43.7)	0.253
Courses on coping with the illness	41 (48.8)	19 (26.8)	**0.008**
Seminars or workshops	31 (36.9)	13 (18.6)	**0.019**
Leaflets/brochures	17 (20.2)	32 (45.1)	**0.001**
Educational offers for the family	29 (34.5)	8 (11.4)	**0.001**
Others^††^	12 (14.3)	7 (10.1)	0.472
No interest	5 (6.0)	9 (12.9)	0.166

**Table 4:** Questions concerning educational and support programs. Data are presented as frequency and valid percent (%) or as mean ± standard deviation.

Variable	US	Germany	*p* value
*Topics for educational and support programs the patients are interested in (multiple answers possible)*	(*N* = 84)	(*N* = 68)	
Communicating with other people	63 (75.0)	36 (52.9)	**0.006**
Relaxation	37 (44.0)	25 (36.8)	0.409
Nutritional advice	43 (51.2)	31 (45.6)	0.518
Stress management	51 (60.7)	23 (33.8)	**0.001**
Exercise	44 (52.4)	31 (45.6)	0.420
Health care bureaucracy/financial issues	30 (35.7)	23 (33.8)	0.865
Coping with daily hassles	31 (36.9)	21 (30.9)	0.493
Others	20 (23.8)	16 (24.2)	1.000
*Type of program the patients are interested in (multiple answers possible)*	(*N* = 84)	(*N* = 61)	
Internet-based program	75 (89.3)	46 (75.4)	**0.040**
In-person seminars: weekend meetings	39 (46.4)	21 (34.4)	0.173
In-person seminars: meetings during the week	30 (35.7)	13 (21.3)	0.068
*Duration of the program*	(*N* = 84)	(*N* = 60)	
Several hours	36 (42.9)	22 (36.7)	0.494
Entire day	11 (13.1)	10 (16.7)	0.634
Entire weekend	2 (2.4)	2 (3.3)	1.000^a^
Flexible timing (available through internet courses only)	35 (41.7)	26 (43.3)	0.866
*How far would the patients travel to attend such an in-person seminar?*	(*N* = 76)	(*N* = 48)	
Miles^b^	168.0 ± 468.01	46.9 ± 49.54	**0.027**
*How many patients would be willing to pay for a support program?*	(*N* = 84)	(*N* = 59)	
Yes	51 (60.7)	31 (52.5)	0.391
No	33 (39.3)	28 (47.5)	
*How much would the patients be willing to pay for a support program?*	(*N* = 48)	(*N* = 30)	
Dollar	150.9 ± 243.35	100.7 ± 196.31	0.501
Euro	137.0 ± 220.81	91.3 ± 178.13	0.501

^a^Fisher's exact test. ^b^1 mile = 1.609 kilometers.

## Data Availability

The data used to support the findings of this study are available from the corresponding author upon request.
